# Assessing executive functions in post-stroke aphasia—utility of verbally based tests

**DOI:** 10.1093/braincomms/fcac107

**Published:** 2022-04-26

**Authors:** Rahel Schumacher, Ajay D. Halai, Matthew A. Lambon Ralph

**Affiliations:** 1 MRC Cognition and Brain Sciences Unit, University of Cambridge, 15 Chaucer Road, Cambridge CB2 7EF, United Kingdom; 2 Department of Neurology, Inselspital, Bern University Hospital, and University of Bern, 3010 Bern, Switzerland

**Keywords:** aphasia, executive functions, neuropsychological test, stroke, voxel-based correlational methodology

## Abstract

It is increasingly acknowledged that, often, patients with post-stroke aphasia not only have language impairments but also deficits in other cognitive domains (e.g. executive functions) that influence recovery and response to therapy. Many assessments of executive functions are verbally based and therefore usually not administered in this patient group. However, the performance of patients with aphasia in such tests might provide valuable insights both from a theoretical and clinical perspective. We aimed to elucidate (i) if verbal executive tests measure anything beyond the language impairment in patients with chronic post-stroke aphasia, (ii) how performance in such tests relates to performance in language tests and nonverbal cognitive functions, and (iii) the neural correlates associated with performance in verbal executive tests. In this observational study, three commonly used verbal executive tests were administered to a sample of patients with varying aphasia severity. Their performance in these tests was explored by means of principal component analyses, and the relationships with a broad range of background tests regarding their language and nonverbal cognitive functions were elucidated with correlation analyses. Furthermore, lesion analyses were performed to explore brain–behaviour relationships. In a sample of 32 participants, we found that: (i) a substantial number of patients with aphasia were able to perform the verbal executive tests; (ii) variance in performance was not explained by the severity of an individual’s overall language impairment alone but was related to two independent behavioural principal components per test; (iii) not all aspects of performance were related to the patient’s language abilities; and (iv) all components were associated with separate neural correlates, some overlapping partly in frontal and parietal regions. Our findings extend our clinical and theoretical understanding of dysfunctions beyond language in patients with aphasia.

## Teaser

Schumacher *et al.* report that, even in chronic post-stroke aphasia, verbal executive tests capture more than patients’ language impairment. Both language and executively related principal components are derived from the patients’ performance, each having separable neural correlates. These results extend the clinical and theoretical understanding of dysfunctions beyond language in aphasia.

## Graphical Abstract

**Graphical abstract:**
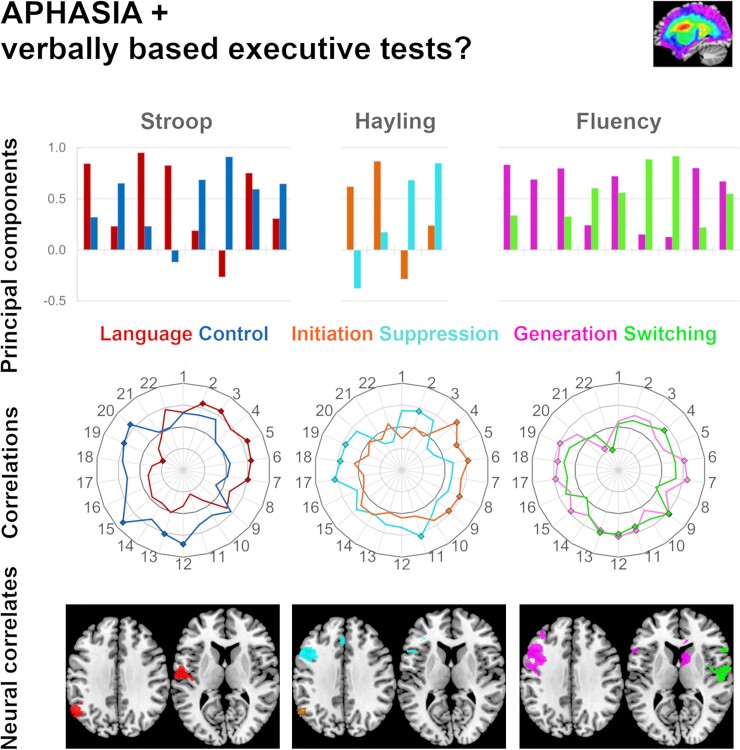

## Introduction

It is increasingly acknowledged that, often, patients with post-stroke aphasia not only have language impairments but also deficits in other cognitive domains (e.g. executive functions)^1–3^ that influence recovery and response to therapy.^4–10^ A variety of tests measuring different aspects of executive functions have been developed, but many commonly used tests contain linguistic stimuli and require speech output. Therefore, they are usually not administered in patients with aphasia. While there are good reasons to be cautious when administering verbally based tests to patients with aphasia, not administering such tests might also be a missed opportunity to gain a better understanding of some of the specific difficulties these patients face with respect to language processing and executive skills. Furthermore, given that the interrelations between language and executive functions remain a matter of interest and debate,^11–13^ performance of patients with aphasia on verbal executive tests might provide valuable insights, both from a theoretical and a clinical perspective.

We recently demonstrated that nonverbal attention and executive deficits in chronic stroke aphasia are multidimensional and independent of deficits to specific language domains.^3^ We not only found that a considerable number of patients showed impaired performance in a battery of nonverbal tests of attention and executive functions but also that the variance underlying performance in language and nonverbal tests was captured by six orthogonal (three language and three nonverbal) components. This result suggests that patients have variable combinations of verbal and non-verbal deficits, all of which should be assessed (and ideally targeted) in a multidisciplinary therapeutic setting.

In the current study, we investigated the same patients’ performance in standardized verbal executive assessments. The broad range of background test data regarding their language and nonverbal cognitive functions allowed us to ask the following key questions: (i) Do verbal executive tests measure anything beyond the language impairment in this patient group? (ii) How does performance in such tests relate to performance in language and non-verbal cognitive tests? (iii) What are the neural correlates associated with performance?

## Materials and methods

### Participants

The same 38 participants reported in a previous study from our group^3^ were recruited for the present study. Only participants who were able to generate at least one correct word in the fluency tests are considered in this analysis, reducing the sample to 32 (11 female, 21 male; see Table 1 for more details). All participants had a single left-hemispheric stroke (ischaemic or haemorrhagic) at least 1 year before assessment and neuroimaging and had no additional significant neurological conditions and no contraindications for MRI. They were pre-morbidly right-handed native English speakers with normal or corrected-to-normal vision. All had been diagnosed with aphasia, but no restrictions were applied regarding the type of aphasia or the severity. Informed consent was obtained from all participants prior to participation, in line with the Declaration of Helsinki and as approved by the local NHS ethics committee.

**Table 1:** Descriptive statistics of performance in the verbal executive tests and patient characteristics

	All patients with available data (*n* = 32)	Subgroups		Comparison between subgroups
		Patients without data on Hayling and/or Stroop (*n* = 15/10)	Patients with data on all verbal executive tests (*n* = 17)	
Fluency (*n* = 32)				
Boy’s names generated	8.5 ± 5.1 (0–19, 84.4%^a^)	5.2 ± 4.1 (0–14, 93.3%^a^)	11.4 ± 4.1 (7–19, 76.5%^a^)	<0.001
Boy’s names accuracy	92.4 ± 19.8 (0–100, n/a)	85.5 ± 27.5 (0–100, n/a)	98.5 ± 3.6 (87–100, n/a)	0.089
Animals generated	7.5 ± 5.0 (0–18)	4.4 ± 3.8 (0–15)	10.2 ± 4.4 (5–18)	<0.001
Animals accuracy	84.3 ± 23.5 (0–100, n/a)	75.4 ± 31.1 (0–100, n/a)	92.2 ± 9.3 (69–100, n/a)	0.061
Fruit/Furniture generated	6.8 ± 3.4 (0–15, 72%)	4.2 ± 2.4 (0–8, 87%)	9.1 ± 2.5 (5–15, 59%)	<0.001
Fruit/Furniture accuracy	84.1 ± 25.6 (0–100, n/a)	75.1 ± 34.3 (0–100, n/a)	92 ± 10.2 (70–100, n/a)	0.084
Fruit/Furniture switches	78.6 ± 31.5 (0–100, 69%)	64.8 ± 40.0 (0–100, 93%)	90.8 ± 13.9 (57–100, 47%)	<0.05
Letter (mean S, P) generated	3.2 ± 2.4 (0–9.5, 93.8%)	2.0 ± 2.0 (0–6, 100%)	4.2 ± 2.3 (1.5–9.5, 88.2%)	<0.01
Letter (mean S, P) accuracy	73.5 ± 29.4 (0–100, n/a)	57.6 ± 34.8 (0–100, n/a)	87.5 ± 13.2 (67–100, n/a)	<0.01
Hayling (*n* = 27)				
Initiation RT	2.4 ± 1.6 (0.3–5.8, 70%)	2.4 ± 1.6 (0.7–5.6, 90%)	2.3 ± 1.7 (0.3–5.8, 58%)	n.s
Initiation accuracy	83.7 ± 16.1 (47–100, n/a)	81.3 ± 16.7 (47–100, n/a)	85.4 ± 16.0 (47–100, n/a)	n.s
Suppression RT	7.8 s ± 6.6 (1.9–28.9, 52%)	10.2 ± 8.5 (2–28.9), 60%)	6.4 ± 4.8 (1.9–19.7, 47%)	n.s
Suppression accuracy	77.3 ± 24.3 (7–100, 4%)	72.9 ± 26.4 (7–93, 0%)	79.6 ± 23.5 (33–100, 6%)	n.s
Stroop (*n* = 17)				
Naming RT	83.8 s ± 38.4 (35–169, 94%)			
Naming accuracy	94.2 ± 6.9 (74–100, 53%)			
Reading RT	59.3 s ± 28.9 (22–120, 88%)			
Reading accuracy	97.2 ± 3.2 (88–100, 41%)			
Interference RT	188.7 s ± 90.3 (77–356, 94%)			
Interference accuracy	90.1 ± 9.4 (66–100, 29%)			
Flexibility RT	168.8 s ± 72.1 (69–300, 71%)			
Flexibility accuracy	88.8 ± 10.9 (60–100, 29%)			
Patient characteristics				
Age	63.7 ± 11.9 (45–88)	70.1 ± 9.1 (52–84)	58 ± 11.4 (45–88)	<0.01
Education	12.5 ± 2.7 (9–19)	11.8 ± 1.7 (10–17)	13.2 ± 3.2 (9–19)	n.s.
Lesion volume	14829 ± 10585 (175–37907)	21135 ± 11643 (4879–37907)	9266 ± 5322 (175–18948)	<0.01
Impairment verbal	61.4 ± 20.8 (21.43–100)	77.6 ± 14.8 (50–100)	47.1 ± 13.6 (21.43–71.43)	<0.001
Impairment nonverbal	35.5 ± 19.7 (6.25–87.5)	47.1 ± 19.4 (18.75–87.5)	25.2 ± 13.7 (6.25–43.75)	<0.001

Numbers indicate mean ± SD (range, % of patients with impaired scores—if applicable); accuracy in percent; RT in seconds. Comparisons between subtests show *P*-values of independent sample *t*-tests comparing the respective means of the two subgroups. Impairment verbal/nonverbal, percentage of impaired performance in the verbal and nonverbal background tests; RT, reaction time; n/a, norm data not available.

^a^ Sum of boy's names and animals taken for norm data comparison.

### Neuropsychological assessments

Three widely used types of standardized verbal executive tests were administered: verbal fluency, Hayling and Stroop. Fluency tests comprised category fluency (animals, boys names and switching between fruit and furniture) from the Delis–Kaplan Executive Function System (D-KEFS)^14^ and letter fluency (S, P) from the Addenbrooke’s Cognitive Examination-Revised (ACE-R)^15^ and the Comprehensive Aphasia Test (CAT),^16^ respectively. Participants were asked to generate as many words as possible within 1 minute, yielding a measure of correct words, rule breaks and repetitions for each subtest, as well as number of realized switches for fruit/furniture. The Hayling test^17^ consists of two subtests, each containing 15 sentences that have the last word missing. The sentences are read aloud by the test administrator and participants are asked to say one word which should complete the sentence in a meaningful way (initiation subtest) or which should not have any meaningful relation to the sentence (suppression subtest). Usually, reaction times (and errors in the suppression subtest) are summed and transformed into a scaled score. As the patients in our sample showed errors and omissions in both subtests, we report the mean reaction times of correctly solved items, alongside the number of omissions and errors (categorized following the handbook for the suppression subtest). The four subtests of the Colour-Word-Interference test (Stroop) of the D-KEFS^14^ were administered, requiring participants to (i) name coloured squares, (ii) read colour words printed in black, (iii) name the colour of the ink of colour words printed in an incongruent colour, and (iv) switch between naming the colour of the ink and reading the word depending on the presence or absence of a frame around the word. All subtests contained the colours red, green and blue and comprised 50 stimuli each, yielding measures of time to complete and number of errors. The test was not administered if it seemed too difficult, based on clinical judgment or performance in the practice items of the first subtest. Furthermore, it was abandoned at participants’ request or if a participant took longer than three minutes to complete the first subtest. Where applicable, comparisons to normative data are given, age-corrected if available. Performance was considered as at least mildly to moderately impaired if it was more than 1.5 SD below the mean (i.e. a T-score below 35, a percentile rank below 6 or a scaled score of 5 or lower).^18^ In addition, comprehensive verbal and non-verbal background testing was available, as reported in previous papers of our group.^3,19–21^ The language-based tests included the following: subtests 1, 2, 8 and 9 from the Psycholinguistic Assessments of Language Processing in Aphasia^22^; word-to-picture matching, naming, and Camel and Cactus Test (CCT) from the 64-item Cambridge Semantic Battery^23^; the Boston Naming Test^24^; a synonym judgement test;^25^ the spoken sentence comprehension task from the CAT^16^; forward and backward digit span^26^; and the Cookie Theft picture description task from the Boston Diagnostic Aphasia Examination.^27^ The nonverbal tests included the following: Alertness, GoNoGo, Divided Attention, and Distractibility subtest from the Test of Attentional Performance^28^; the subtests Design Fluency and Trail Making (parts 2–4) from the D-KEFS^14^ a computerized version of the Tower of London (by Schuhfried)^29^; the Kramer test^30^; the Raven’s Coloured Progressive Matrices^31^; and the Brixton test.^17^ Performance on these tests also served to compute the severity of an individual's language/nonverbal impairment (given as percentage of impaired scores). For example, if a patient’s performance was impaired in 9 f 10 administered language tests, their language impairment (or severity) would be given as 90%.

### Statistical analysis

Principal component analyses with varimax rotation were computed (using IBM SPSS 22.0) for each verbal executive test separately, to elucidate whether performance in a given test would be best explained by one or more components (i.e. reflect the multiple cognitive features built into the assessment design or, instead, simply reduce to a single dimension of variation as one would expect if performance in this clinical group solely reflected their aphasia and not independent variation in non-language impairments). To ease interpretation, we ensured that a higher score indicated better performance for all measures. To this end, reaction time measures were inverted, and accuracy rates were computed. All components with eigenvalues ≥1 were extracted and then varimax rotated, yielding orthogonal and interpretable components. Spearman correlations were computed to explore the relationship between component scores and the background measures. These correlations were computed on an overall and a specific level. The former comprised correlations with overall verbal and nonverbal severity of a patient’s impairment, respectively. The latter comprised correlations between component scores and performance in specific background tests.

### Neuroimaging data acquisition and analysis

High resolution structural T1-weighted MRI scans were acquired on a 3.0 Tesla Philips Achieva scanner (Philips Healthcare, Best, The Netherlands) using an 8-element SENSE head coil. A T1-weighted inversion recovery sequence with 3D acquisition was employed, with the following parameters: TR (repetition time): 9.0 ms, TE (echo time)    3.93 ms, flip angle = 8°, 150 contiguous slices, slice thickness = 1 mm, acquired voxel size 1.0 × 1.0 ×1.0 mm, matrix size 256 × 256, field of view = 256 mm, TI (inversion time) = 1150 ms, SENSE (sensitivity encoding) acceleration factor 2.5, and total scan acquisition time = 575 s.

**Figure 1:**
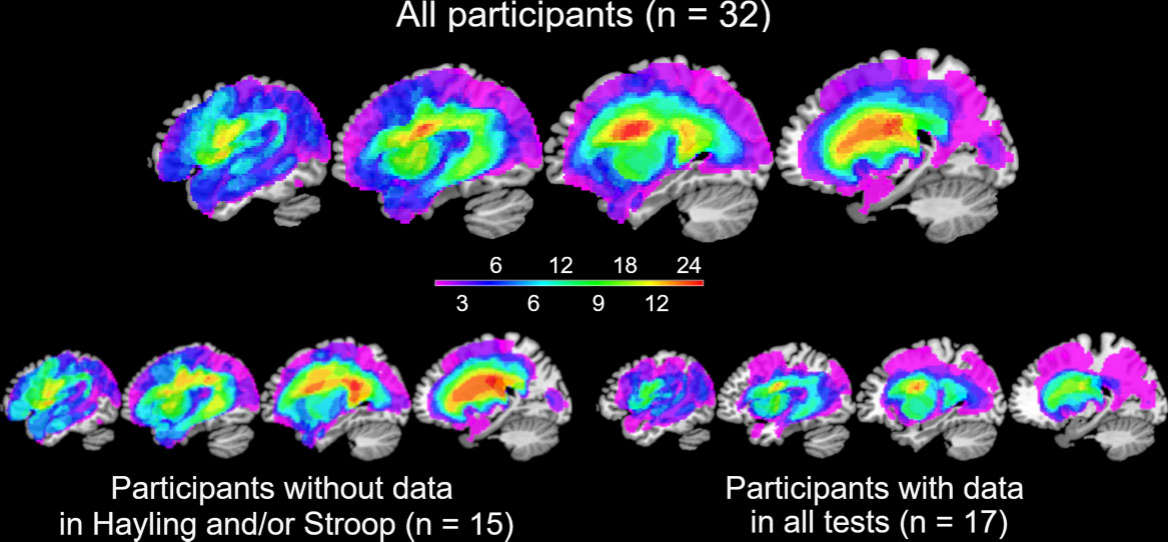
**Overlap maps of the patients’ lesions.** The slices correspond to MNI coordinates of *x* = −50, −40, −30, −20, from left to right. The figures are thresholded at the maximum overlap (*n* = 25 in full sample, *n* = 14 in subsamples).

Structural MRI scans were pre-processed with Statistical Parametric Mapping software (SPM8: Wellcome Trust Centre for Neuroimaging, http://www.fil.ion.ucl.ac.uk/spm/). The images were normalized into standard Montreal Neurological Institute (MNI) space using a modified unified segmentation-normalization procedure optimized for focal lesioned brains.^32^ Data from all participants with stroke aphasia and all healthy controls were entered into the segmentation—normalization. Images were then smoothed with an 8 mm full-width half-maximum Gaussian kernel and used in the lesion analyses described below. The lesion of each patient was automatically identified using an outlier detection algorithm, compared with healthy controls, based on fuzzy clustering. The default parameters were used apart from the lesion definition ‘U-threshold’, which was set to 0.5 to create a binary lesion image. We modified the U-threshold from 0.3 to 0.5 after comparing the results obtained from a sample of patients to what would be nominated as lesioned tissue by an expert neurologist. The images generated were used to create the lesion overlap map in Fig. 1.

The normalized and bias-corrected T1-weighted images were used to determine the brain regions where tissue concentration correlated with behaviour using a voxel-based correlational methodology (VBCM),^33^ a variant of voxel-lesion symptom mapping,^34^ in which both the behaviour and signal intensity measures are treated as continuous variables (conducted in SPM12). For the neural correlate analysis, we are assuming that lower T1-weighted intensity is related to tissue damage or atrophy. For each verbal executive test separately, the participants’ component scores (from the principal components analysis) were entered simultaneously into a VBCM analysis. The resulting lesion clusters thus account for the unique variance of a component. The applied threshold at voxel-level was *P* < 0.001 and at cluster-level *P* < 0.001, unless noted otherwise. The anatomical labels for the clusters were determined using the Harvard–Oxford atlas for grey matter and on the John Hopkins white matter atlas for white matter tracts.

### Data availability

The data that support the findings of this study are available from the corresponding authors, upon reasonable request.

## Results

### Descriptive statistics of performance and influence of patient characteristics

**Figure 2:**
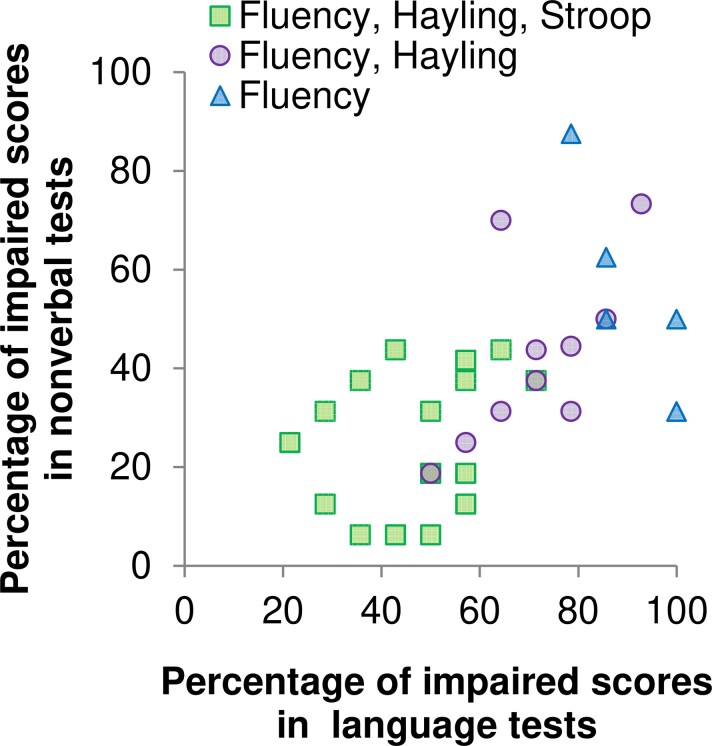
Distribution of patient subgroups as a function of their overall impairment in nonverbal versus language tests.

Out of the 38 patients, 32 had a minimum score of one correctly generated word in any one of the fluency subtests. Twenty-seven patients of those 32 completed both Hayling subtests (incomplete datasets in two patients where the suppression subtest was not administered due to high error rates and need for several sentence repetitions in the initiation subtest; test not administered in three patients based on clinical judgment), and 17 completed all Stroop subtests (not possible in one patient because of colour blindness; abandoned during or after first part in nine patients due to difficulties with colour naming; not administered in five patients based on clinical judgement). Fig. 2 plots the severities of the nonverbal and language impairment of each individual and shows whether it was possible to completely administer the Stroop and/or Hayling Test in addition to the fluency tasks.

Table 1 summarizes the performance in all verbal executive tests and indicates how many participants showed impaired performance compared to norm data (if available). Patient characteristics were compared between the subgroup who completed all verbal executive assessments versus the rest of the sample. Differences between the groups were found on all background characteristics with the exception of education.

### Principal component analyses and correlations

To elucidate if one or more components explained the patients’ performance in the three different tests of executive function, separate principal component analyses were performed. In addition, to better understand the interrelations in a wider context, performance in the verbal executive tests was correlated with performance in the language and nonverbal background measures.

#### Stroop

**Figure 3:**
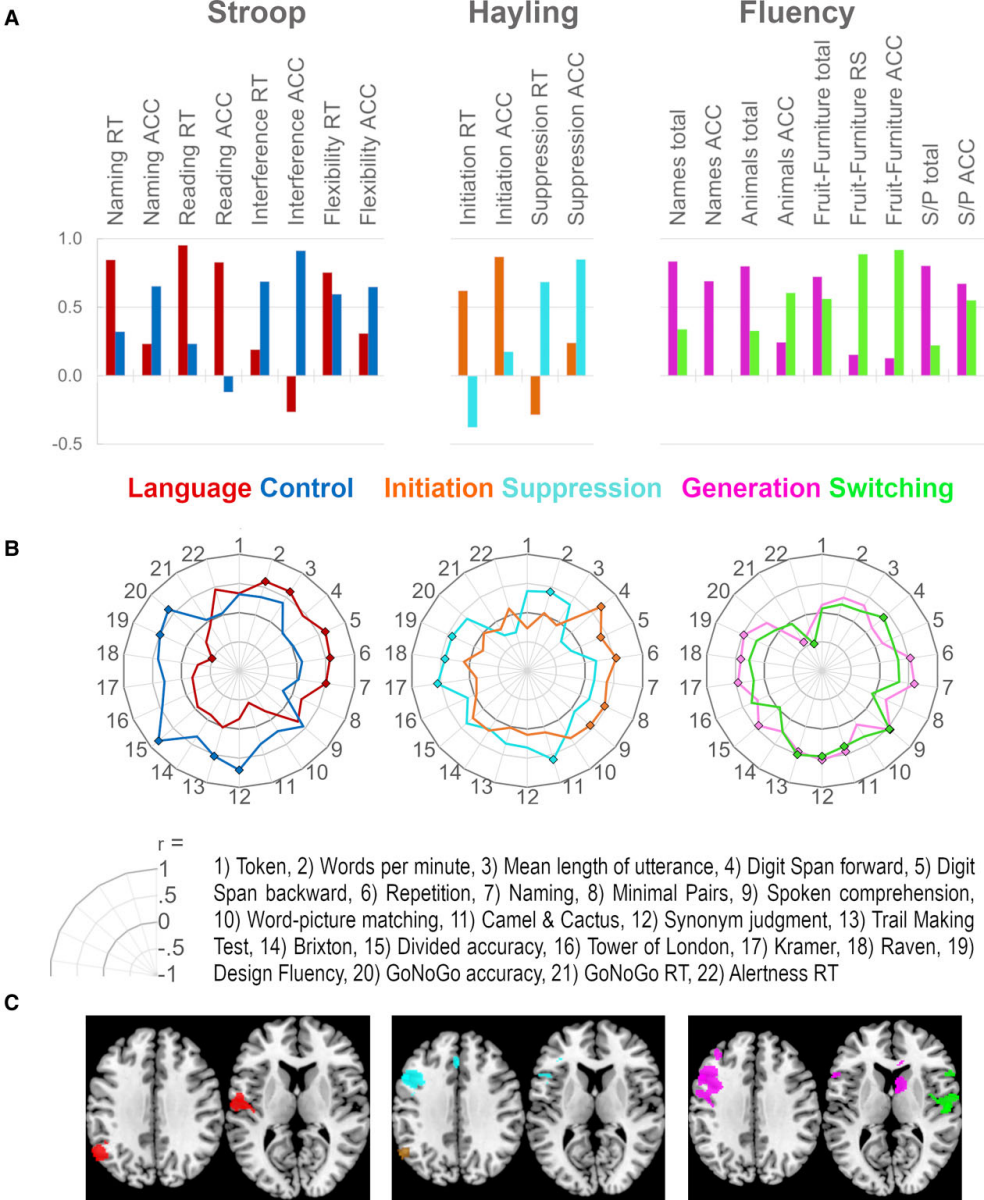
**Component loadings, correlations between factor scores and background tests and structural correlates associated with verbal executive test performance.** (**A**) The bars represent the loadings of the individual task measures on the components extracted by means of principal components analyses. Three separate analyses were conducted which yielded two components each. The component interpretation is given underneath, and the colour-coding is maintained throughout the figure. Loadings < 0.1 are not depicted. ACC, accuracy; RT, reaction time; RS, realized switches; S/P, letters S or P. (**B**) Radar plots depicting the correlations between an individual’s factor scores on each component and their performance in language and nonverbal tests. The centre represents a correlation coefficient of *r* = −1 and the outer ring a correlation of *r* = 1 (increments of 0.5 in between). Significant correlations are indicated with a square. Example: A high score on the Control component of the Stroop was significantly associated with a high accuracy in the GoNoGo test. (**C**) Axial slices showing the structural correlates associated with each component (MNI space *z* = 35 and 10, left is left). See Table 3 and Figure 4 for more detailed information on the results of the VBCM analyses.

The principal component analysis [*n* = 17, Kaiser–Meyer–Olkin (KMO) = 0.634, Bartlett's sphericity test *P* < 0.001] revealed two components, accounting for 72.6% of the variance. The loadings of the reaction time and accuracy measures of each subtest on the two components are shown in Fig. 3A. The first component explained 39.2% and was interpreted as capturing ‘language’ because naming and reading speed loaded highest on this component. Importantly, there was clear evidence that this task was still sensitive to executive skill level in this group of patients with aphasia. Specifically, the second component explained 33.5% and was interpreted as capturing ‘control’ because the number of errors in the interference condition (i.e. the central design feature of the Stroop) loaded highest on this component. When correlated with the severity of the language and nonverbal impairment (as percentage of impaired performance in the respective background tests), a high score on the Language component was associated with a low severity of the language impairment, while a high score on the Control component was associated with a low severity of the nonverbal impairment, as shown in Table 2. These distinctive correlations with overall severity were further underlined by different patterns of significant correlations with specific tests, as shown in Fig. 3B.

**Table 2:** Pairwise Pearson correlations within and between component scores and severity of patient's language and nonverbal impairment

		Impairment		Hayling		Fluency	
		verbal	nonverbal	Initiation	Suppression	Generation	Switching
Stroop	Language	−0.564*	−0.224	0.446^#^	−0.060	0.077	0.134
	Control	−0.344	−0.616**	0.039	0.335	0.387	0.220
Hayling	Initiation	−0.450*	−0.172			0.056	0.433*
	Suppression	−0.304	−0.313			0.504**	−0.302
Fluency	Generation	−0.438*	−0.378*				
	Switching	−0.418*	−0.415*				

Note: ^#^*P* < 0.1, *P < 0.05, **P < 0.01, two-sided.

#### Hayling

Again, there was clear evidence that the features of executive control built into the test were present in the data from patients with aphasia. The principal component analysis (*n* = 27, KMO = 0.448, Bartlett's sphericity test *P* = 0.425) revealed two components, accounting for 65.6% of the variance. The first component explained 34.1% and reflected performance in the suppression subtest, while the second component explained 31.6% and reflected performance in the initiation subtest (as depicted in Fig. 3A). The Suppression component did not correlate significantly with any of the two overall severity measures, but high scores on the Initiation component were associated with low severity of the language impairment (see Table 2). Correlations on the specific test level revealed that higher scores on the Initiation component were associated with better performance in the language tests repetition and digit span, whereas high scores on the Suppression component was associated with better performance in the Kramer as well as the CCT, as shown in Fig. 3B.

A KMO below 0.5 indicates suboptimal fit for this type of analysis. The subsequent analyses reported below (correlations, VBCM), however, yielded very similar results for the Hayling test whether the raw data of the four measures were included or the component scores derived from the principal component analysis. Thus, for simplicity and consistency, we maintain the same data-driven measures across all verbal executive tests.

#### Fluency

As observed with the previous tests, here again, we found evidence that performance in fluency tasks was based on more than one component. The principal component analysis (*n* = 32, KMO = 0.832, Bartlett's sphericity test *P* < 0.001) revealed two components, accounting for 71.5% of the variance. The first component explained 39.3% and was interpreted as ‘generation’ because the number of generated items in all conditions loaded highest on this component. The second component explained 32.2% and was interpreted as ‘switching’ because the task where participants were asked to switch between saying fruit and furniture loaded highest on that component. Higher scores on both fluency components were associated with lower severity of the language and of the nonverbal impairment (see Table 2). The correlations between the individual scores on the two components and specific tests varied slightly, but no clearly different pattern was observed (see Fig. 3B).

### Correlations between verbal executive tests

The ability of these ‘verbal’ tests to detect and grade variation in executive skill was further underlined by examining their intercorrelations. There were two significant correlations between the factor scores extracted from verbal executive tasks. As detailed in Table 2, high scores on the Hayling Suppression component were associated with high scores on the Fluency Generation component, while high scores on the Hayling Initiation component were associated with high scores on the Fluency Switching component. The association between high scores on the Hayling Initiation and Stroop Language component reached borderline statistical significance.

### Structural correlates

**Figure 4:**
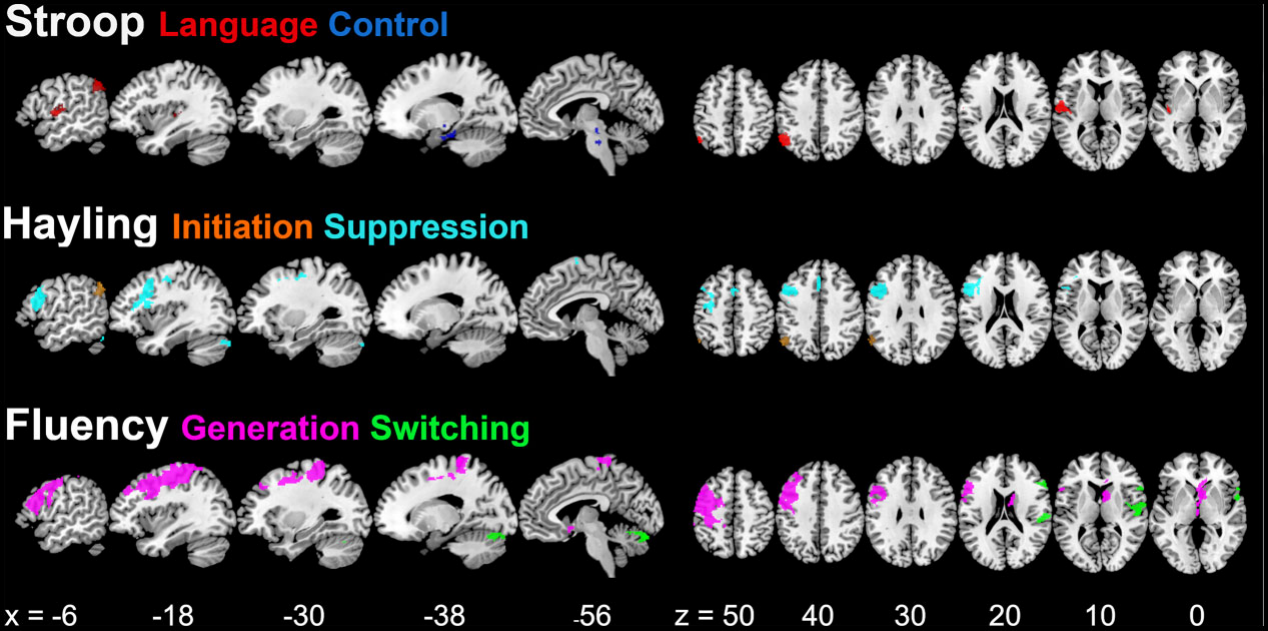
**Structural correlates associated with the verbal executive test components.** Clusters were obtained by applying a voxel-level threshold of *P* ≤ 0.001, and a family-wise error correction of *P* ≤ 0.001 on cluster-level (apart from Hayling Initiation where *P* ≤ 0.005 and *P* < 0.05, respectively, was applied). The slices are in neurological convention (left is left) and the coordinates shown are in MNI-space.

To elucidate the associations between a patient's lesion and their performance in the verbal executive tests, we performed separate VBCM analyses for each test. In each of the three analyses, an individual's scores on both components derived from the principal components analysis were simultaneously included as continuous variables, thus yielding clusters that explain variance uniquely associated with each component. Significant clusters emerged for all measures, as depicted in Fig. 4 and detailed in Table 3

**Table 3:** Clusters and peaks associated with the component scores of the verbal executive tests

Component	Extent	Location	L/R	Z	*x*	*y*	*z*
Stroop Language	372	Angular gyrus	L	4.24	−58	−58	42
		Angular gyrus	L	4.08	−50	−54	40
		Lateral occipital cortex sup	L	4.00	−48	−60	38
	346	Central operculum cortex	L	4.65	−58	−6	6
		Central operculum cortex	L	4.27	−58	−14	12
		Heschls gyrus	L	3.71	−40	−18	4
Stroop Control	223	Hippocampus	L	3.90	−20	−14	−18
		Brain Stem		3.90	−4	−22	−14
		Brain Stem		3.76	−10	−32	−20
Hayling Initiation	354	Supramarginal gyrus pos	L	3.17	−62	−48	26
		Angular gyrus	L	3.13	−54	−58	34
		Lateral occipital cortex sup	L	3.10	−54	−62	44
Hayling Suppression	1693	Inferior frontal gyrus p ope	L	4.72	−48	14	24
		Middle frontal gyrus	L	4.25	−48	10	38
		Middle frontal gyrus	L	4.21	−52	16	40
	312	Paracingulate gyrus	R	4.94	2	16	46
		Paracingulate gyrus	L	3.56	−2	32	36
		Supplementary motor cortex		3.48	0	8	66
	303	Cerebellum	L	4.38	−40	−78	−28
		Cerebellum	L	4.38	−44	−80	−28
		Cerebellum	L	4.05	−52	−66	−24
Fluency Generation	5816	Postcentral gyrus	L	4.99	−48	−38	60
		Postcentral gyrus	L	4.94	−38	−34	54
		Postcentral gyrus	L	4.89	−44	−34	58
	1006	Caudate	R	4.48	6	8	6
		Caudate	R	4.47	10	8	4
		Subcallosal cortex	R	4.38	2	8	−14
Fluency Switching	1385	Cerebellum	R	6.12	14	−78	−24
		Cerebellum	R	5.53	38	−54	−28
		Cerebellum	R	5.48	30	−68	−22
	541	Parietal operculum cortex	R	5.18	60	−22	16
		Central operculum cortex	R	4.78	54	−10	10
		Precentral gyrus	R	4.49	62	−2	16
	326	Central operculum cortex	R	4.78	56	6	0
		Planum polare	R	4.72	52	6	−6
		Inferior frontal gyrus p ope	R	4.52	62	20	16

The three highest peaks per cluster are given with coordinates in MNI space. A more detailed table is provided in the Supplementary material.

L/R, left or right side of the brain; p ope, pars opercularis; p tri, pars triangularis; pos, posterior; sup, superior.

For the Stroop test, the Language component was associated with two clusters. The posterior cluster comprised parts of the angular gyrus and bordering superior lateral occipital cortex, and the anterior cluster included structures around the insula. The cluster found for the Control component included medial temporal areas and extended into the brain stem. The Initiation component of the Hayling test was, similarly to the Stroop Language component, associated with a posterior cluster that included parts of the angular gyrus, superior lateral occipital cortex and a portion of the supramarginal gyrus. The Suppression component in turn was mainly associated with a frontal cluster in the middle and inferior frontal gyrus, in addition to a small frontal midline cluster in the anterior cingulate, and a further small cluster in the cerebellum. The Generation component of the fluency tests was associated with a large cluster, mainly including frontal areas (especially middle frontal gyrus) but also extending into the parietal lobe. Moreover, bilateral midline structures were associated with this component. The clusters associated with the Switching component included right fronto-temporo-insular as well as cerebellar structures.

## Discussion

It is increasingly recognized that post-stroke aphasia can co-occur with variable levels of executive deficits, which can impact on everyday abilities, recovery success and therapy efficacy.^1–10^ Accordingly, it is imperative to increase our understanding of the nature and types of executive impairments in this clinical population. The literature on executive skill is, however, dominated by verbally based assessments. These tests are typically avoided in aphasia clinical practice and research studies on the untested assumption that the results of such assessments will be irretrievably contaminated by the patients’ language impairment. We addressed and overturned this assumption through a systematic evaluation of verbal executive tests across a sample of patients who spanned the full range of aphasia severity. Our analysis of patients’ performance in these tests revealed that: (i) a substantial number of patients with aphasia were able to perform the tasks; (ii) variance in performance was not explained by the severity of an individual's overall language impairment alone but was related to two independent behavioural principal components per test; (iii) not all aspects of performance were related to the patients’ language impairment and instead reflected the core executive feature that each test was designed to probe; and (iv) all components were associated with separate neural correlates, some overlapping partly in left frontal and parietal regions.

Given that all patients studied by our group were diagnosed with aphasia, it was expected that verbal executive test administration would not be feasible in everyone. Of the patients who generated at least one correct word in a fluency test (leading to inclusion in the current analyses), approximately 85% completed the Hayling and 50% also the Stroop test. Patients in whom complete administration of all verbal executive tests was not possible were older and more severely affected. While the investigation and thus the following discussion focuses on the data we were able to collect, more general ramifications for diagnosis and therapy will also be considered.

Our first two aims were to elucidate whether verbal executive tests measure anything beyond the language impairment in this patient group and how verbal executive test performance relates to the patients’ other language and nonverbal cognitive abilities. Finding two orthogonal components for each test indicates that performance cannot be explained by the overall severity of an individual’s language impairment alone (which would result in a single principal component that correlated with the patients’ aphasia severity). More detailed insights were revealed by the correlation analyses, which yielded different patterns of correlations with the overall language or nonverbal impairment and with performance in the specific background tests for the Stroop, Hayling and fluency tests, respectively.

For the Stroop test, the findings were most clear-cut. As one might expect, more severe language impairments were associated with lower scores on the Stroop Language component. Importantly, beyond language, the results showed that more severe nonverbal impairments were associated with lower scores on the Stroop Control component. Intriguingly, naming performance was related not only to the Language component but also the Control component. This makes sense as the task requires a form of blocked-cyclical naming. Having to repeatedly select the correct item among a limited set of previously activated and semantically related items increases control demands, as has previously been shown.^35,36^ In addition, we found a strong association between low performance in a nonverbal test of divided attention and low scores on the Control component. This is, in our view, particularly interesting and extends a recent observation in healthy controls, whose ability to avoid distracting information in a semantic decision task was disrupted if they had to divide their attention.^37^ The significant correlation in our data would fit with the authors’ speculation of a shared cognitive resource for distractor inhibition and divided attention. Hence, our patients’ difficulties in semantic selection as well as in dividing attention might be linked to the same domain-general control impairment. In fact, the Stroop task is sometimes conceptualized as a test of attention,^38,39^ which further underlines that some aspects of executive function and attention overlap.

The two components found for the Hayling test aligned with its two intended subtests (Initiation, Suppression), confirming dissociations found in other patient groups.^40^ While patients with more severe language impairments had lower scores on the Initiation component (as one might expect in aphasia), no significant correlations between impairment severities and the Suppression component were found. However, correlations with specific background measures revealed that performance in the Suppression component was associated with tests of nonverbal abstraction, reasoning and idea generation, alongside the speed of language production in a connected speech task. Thus, in patients with aphasia, the Hayling's sentence completion subtest seems to be mainly sensitive to language impairments while performance in the suppression subtest is additionally influenced by other aspects of cognition.

In contrast to the Hayling and Stroop tests, fluency tests are more commonly administered in patients with aphasia.^41–44^ The two components derived from the principal component analysis did not separate the semantic and phonemic versions of the tasks, as found previously^45^ but were interpreted as Generation (reflecting the number of correctly produced words) and Switching (reflecting the ability to switch between categories as well as overall accuracy of responses). Both components were significantly, albeit moderately, correlated with both the language and the nonverbal impairments of the patients. In addition, the patterns of correlations on the specific level were similar and included language as well as nonverbal tests for both components.

Considering performance across the verbal executive tests revealed significant correlations between the two components of the Hayling and fluency tests. First, the ability to suppress a prepotent response and instead produce a semantically unrelated word in the Hayling test was associated with the ability to generate many different words in the fluency tasks. In both types of tasks, inhibition (of the meaningful completion or of already generated words) is required alongside idea generation. Intriguingly, factor scores on these two components correlated significantly with the same three nonverbal tests (Kramer, Raven, Design Fluency). These nonverbal tests pose similar demands with respect to inhibition and idea generation but do not necessitate language production.^3^ Second, successful sentence completion in the Hayling test was associated with better switching abilities in the fluency tests. The Switching component of the fluency tests reflected the number of realized switches in the category switching test and the accuracy of responses in other fluency tests; this association might be interpreted as reflecting the efficiency of lexical access across a variety of situations in which the search space is more or less constrained.

Importantly, even though the three types of verbal executive tests are similar in requiring the production of single words, they show different relationships to other tests (language and beyond) and only limited cross-correlations. Therefore, they seem to capture somewhat different aspects of language and executive functions. The multifactorial nature of executive functions aligns with recent research—including the verbal executive tests used in this study— in other patient samples,^46^ in healthy controls,^47–49^ and when using different tasks in patients with aphasia.^12^ Taken together, our behavioural analyses at the group level: (i) formally show that a patient’s performance in verbal executive tests is not solely driven by their language abilities; (ii) converge with our previous research by demonstrating that there can be co-occurring executive problems which are observable not only in nonverbal but also in verbal tasks; and (iii) indicate that every test captures a slightly different aspect of language and executive function.

In what way is this now meaningful for an individual, and, importantly, for clinical practice? First, due to its demands, the Stroop test could only be administered to a limited subset of patients who tended to be less severely impaired and younger. However, within this selection of patients, the test seems to be a very sensitive marker of reduced processing efficiency as all patients, bar one, were slowed in naming coloured squares when compared with normative data. Moreover, given the relationship between the Control component and performance in nonverbal tests of attention and executive functions, it does not seem to be justified to attribute impaired performance in the interference subtest solely to impaired language functions. Thus, in patients with mild-to-moderate aphasia, if an individual’s performance in the interference condition is disproportionately slowed and error-prone compared with naming coloured squares, an additional executive impairment in interference resolution seems highly probable. Similarly, a disproportionately worse performance in the flexibility condition when compared with the first two conditions will point to an issue with cognitive flexibility.

Second, in the Hayling test, while sentence completion in a meaningful or unrelated way was slowed for the majority of patients, the number of suppression errors was increased in only a few. This qualitative observation extends previous research documenting an increased number of suppression errors particularly in patients with right frontal lesions.^50^ Thus, an increased number of suppression errors would also in this patient group be indicative of an executive impairment. Furthermore, different performance patterns between the two subtests will help disentangling difficulties with sentence comprehension/lexical access/word production from difficulties with idea generation/inhibition.

Third, with respect to the fluency tests, our data confirm their usefulness as an indicator for the presence of some cognitive difficulty, but at the same time reveal their limited specificity regarding the source of impaired performance. This explains the lack of consensus as to how such tests should be conceptualized,^42,51,52^ but also justifies their inclusion in screening measures of global cognitive status such as Montreal Cognitive Assessment^53^ and ACE-R.^15^ Yet, difficulties in language- or more general control-related aspects, or both, might lead to similar performance patterns and additional assessments are needed to gain more detailed insights on an individual level.

If test administration is feasible, which generally seems to be the case in patients with mild to moderate aphasias, verbal executive tests can either complement findings based on nonverbal tests or may even be more sensitive and reveal difficulties that would otherwise not have been detected. Gaining a comprehensive understanding of someone’s difficulties and resources is not only informative in itself but also has a potential impact on the level of activity and participation. Importantly, while the relationship between executive function and functional communication—a central aspect of activity and participation in patients with aphasia—has been studied previously,^2,21,54–56^ further research elucidating specific^57^ as well as more complex aspects of communication will be of interest and relevance. For instance, separate lines of research have investigated ‘cognitive communication disorders’ in other patient populations (e.g. traumatic brain injury), in part by means of the same verbal executive tests used here.^58–60^ Connecting these literatures might stimulate interesting future avenues of research.

Instead of encouraging or discouraging the administration of certain tests in certain patients, we would argue for a clinically informed approach to test administration, as well as interpretation. Importantly, rather than pre-emptively deciding that a patient will not be able to do a certain task, it is often more informative to try it, as is underscored by our data. Of course, an individual’s abilities and tolerance to frustration are always important aspects to consider, not to speak of the time available for assessments. Thus, deciding whether one should administer a certain test requires expertise and depends on the question as well as circumstances. When interpreting obtained measures, we would like to stress that, like in any other patient population, the whole profile is key. Therefore, not only measures within a test but across the whole range of neuropsychological tests/domains should be considered in order to understand a patient’s difficulties and resources.

Given that the knowledge of somebody’s difficulties and resources is paramount to planning interventions, we would like to raise one last point. While we have focused on the usefulness of verbal executive assessments, it is also important to consider patients who are (for lack of language abilities) not able to complete such tests. Crucially, the absence of evidence should not be misinterpreted as the absence of difficulties beyond language. Such an approach might lead to underestimation—and therefore ‘under treatment’—of potential difficulties. There is no simple solution to this problem, but we would argue that involving a multidisciplinary team in diagnosis and therapy of these patients might counteract this issue at least to some extent.

Our third aim was to explore the neural correlates associated with performance in the verbal executive tests. Significant, partly overlapping clusters where structural integrity correlated with performance were found for all six behavioural components. The two more language-related components, Stroop Language (colour naming/reading) and Hayling Initiation (sentence completion), were associated with partly overlapping left inferior parietal clusters. This is in line with previous research on language processing, including reading^61^ and sentence comprehension.^62^

The clusters associated with the Generation component of the fluency tasks as well as the Suppression component of the Hayling comprised mainly left frontal areas. They partly overlapped, most notably in the left middle and inferior frontal gyrus, in line with previous research on the Hayling^63^ or fluency tests.^44,64^ This frontal region has been linked to language^65^ as well as the multi-demand-network^66^ (i.e. corresponds to their overlap; maps retrieved from http://web.mit.edu/evlab//funcloc/). Moreover, a recent meta-analysis found the same inferior and middle frontal regions to be involved in semantic control^67^ and the left inferior frontal gyrus in particular is thought to play an important role in concept-level selection of competitors.^68^ Surprisingly, the cluster associated with the Stroop Control component did not contain any frontal areas but was limited to a small part of the medial temporal lobe and brain stem structures, which is difficult to interpret. This finding—or non-finding—might partially be explained by sampling issues. Not only is the sample size considerably smaller than in the other analyses, but the sample is also constrained with respect to lesion size (reducing the probability of overlapping lesions) and with respect to behavioural performance (as patients who, maybe also for reasons to do with the control aspect of interest, are not able to do the task sufficiently well to complete it). However, the fact that no stable correlation can be obtained also means that the impairments observed in the patients who were able to complete it may have somewhat differing neural bases. This is in line with many other studies (e.g.^69,70^) indicating that structures beyond the frontal lobe—as well as their connections—play an important role in executive functioning (for a recent study in aphasia see^71^).

Finally, both fluency components were associated with right fronto-temporal areas. Previous research in healthy participants has shown bilateral activations in fluency tasks,^64^ which is also reflected in patient studies.^43,72^ For instance, in a group of patients with aphasia, category fluency task performance was associated with the bilateral, so called, cingulo-opercular network,^43^ which comprises the areas found in our analysis. Importantly, our approach to map brain structure integrity and behaviour included the whole brain and not only the core ‘affected’ (in this case left) hemisphere. While the primarily lesioned area and its (severed) connections will be the most important source of impaired cognitive performance, secondary structural changes following stroke, associated chronic vascular load or potentially unrelated (for instance age-related) alterations might also affect performance. Finding meaningful right-hemisphere clusters by means of such an approach might thus prompt other research groups to consider similar avenues for future research.

In sum, we demonstrate that verbal executive function tests not only capture different features of patients' language impairments but also reveal information on potential, independent impairments in other aspects of their cognitive functioning. This extends our understanding of dysfunctions beyond language in patients with aphasia and has not only theoretical but also clinical implications.

## Supplementary Material

fcac107_Supplementary_Data

## Abbreviations

D-KEFS =: Delis–Kaplan Executive Function System
KMO =: Kaiser–Meyer–Olkin (test for sampling adequacy)
MNI =: Montreal Neurological Institute
VBCM =: voxel-based correlational methodology
