# Supplementary material for: Assessing executive functions in post-stroke aphasia—utility of verbally based tests
Source: Brain Commun. 2022 Apr 26;4(3):fcac107. doi: 10.1093/braincomms/fcac107 (PMC9118101; doi:10.1093/braincomms/fcac107)
Supplement: fcac107_Supplementary_Data [file fcac107_supplementary_data.pdf]

Supplementary Table 1. Clusters and peaks associated with the component scores of the verbal executive tests.

Component	Ext	Location	L/R	Z	x	y	z
**Stroop Language**	372	Angular gyr	L	4.24	-58	-58	42
		Angular gyr	L	4.08	-50	-54	40
		Lat occ cor sup	L	4.00	-48	-60	38
		Lat occ cor sup	L	3.81	-44	-62	40
		Angular gyr	L	3.81	-46	-52	44
		Supram gyr pos	L	3.48	-52	-48	38
	346	Cent operc cor	L	4.65	-58	-6	6
		Cent operc cor	L	4.27	-58	-14	12
		Heschls gyr	L	3.71	-40	-18	4
		Heschls gyr	L	3.64	-46	-10	4
		Heschls gyr	L	3.43	-38	-24	6
		Postcent gyr	L	3.25	-58	-12	18
**Stroop Control**	223	Hippocampus	L	3.90	-20	-14	-18
		Brain Stem		3.90	-4	-22	-14
		Brain Stem		3.76	-10	-32	-20
		Amygdala	L	3.74	-22	-10	-16
		Hippocampus	L	3.68	-20	-10	-20
		Brain Stem		3.67	-2	-26	-28
		Parahipp gyr pos	L	3.60	-16	-26	-20
**Hayling Initiation**	354	Supram gyr pos	L	3.17	-62	-48	26
		Angular gyr	L	3.13	-54	-58	34
		Lat occ cor sup	L	3.10	-54	-62	44
		Lat occ cor sup	L	3.08	-52	-62	48
		Lat occ cor sup	L	2.95	-60	-62	30
		Lat occ cor sup	L	2.95	-58	-64	34
		Lat occ cor sup	L	2.89	-60	-64	26
		Angular gyr	L	2.84	-62	-60	24
		Angular gyr	L	2.65	-64	-56	22
**Hayling Suppression**	1693	Inf fro gyr p ope	L	4.72	-48	14	24
		Mid fro gyr	L	4.25	-48	10	38
		Mid fro gyr	L	4.21	-52	16	40
		Mid fro gyr	L	4.15	-48	16	38
		Mid fro gyr	L	4.04	-40	16	28
		Precent gyr	L	3.82	-34	-12	50
		Mid fro gyr	L	3.77	-36	10	40
		Inf fro gyr p tri	L	3.59	-36	32	16
		Inf fro gyr p tri	L	3.50	-40	26	18
		Mid fro gyr	L	3.45	-48	16	46
		Mid fro gyr	L	3.26	-32	6	50
		Precent gyr	L	3.25	-42	-8	52
		Inf fro gyr p ope	L	3.24	-40	12	18
		Fro operc cor	L	3.19	-42	18	8
		Precent gyr	L	3.19	-46	-2	38
	312	Paracingulate gyr	R	4.94	2	16	46
		Paracingulate gyr	L	3.56	-2	32	36
		Supp mot cor		3.48	0	8	66
		Supp mot cor	L	3.37	-4	2	70
		Supp mot cor		3.22	0	2	58
	303	Cerebellum	L	4.38	-40	-78	-28
		Cerebellum	L	4.38	-44	-80	-28
		Cerebellum	L	4.05	-52	-66	-24
		Cerebellum	L	3.94	-50	-72	-22
		Cerebellum	L	3.81	-42	-70	-30

Component	Ext	Location	L/R	Z	x	y	z
**Fluency Generation**	5816	Postcent gyr	L	4.99	-48	-38	60
		Postcent gyr	L	4.94	-38	-34	54
		Postcent gyr	L	4.89	-44	-34	58
		Postcent gyr	L	4.47	-26	-34	58
		Postcent gyr	L	4.43	-22	-36	64
		Inf fro gyr p tri	L	4.42	-58	22	24
		Inf fro gyr p ope	L	4.27	-52	18	24
		Postcent gyr	L	4.21	-12	-38	78
		Precent gyr	L	4.20	-50	-10	56
		Postcent gyr	L	4.14	-20	-42	76
		Postcent gyr	L	4.13	-14	-40	62
		Precent gyr	L	4.05	-46	-6	50
		Postcent gyr	L	4.03	-16	-36	70
		Mid fro gyr	L	4.02	-30	36	42
		Precent gyr	L	4.00	-36	-18	44
		Mid fro gyr	L	3.96	-52	12	46
	1006	Caudate	R	4.48	6	8	6
		Caudate	R	4.47	10	8	4
		Subcallosal cor	R	4.38	2	8	-14
		Caudate	L	4.28	-4	8	-8
		Subcallosal cor	R	4.09	2	16	0
		Forceps major		3.89	4	18	4
		Forceps major		3.87	10	32	4
		Caudate	R	3.55	12	8	20
		Thalamus	L	3.52	0	-4	-4
		Caudate	R	3.48	12	12	18
		Thalamus	L	3.40	-2	-14	2
		Forceps minor		3.30	6	28	14
**Fluency Switching**	1385	Cerebellum	R	6.12	14	-78	-24
		Cerebellum	R	5.53	38	-54	-28
		Cerebellum	R	5.48	30	-68	-22
		Cerebellum	R	5.19	32	-62	-24
		Cerebellum	L	4.75	-10	-78	-22
		Cerebellum	L	4.59	-6	-82	-22
		Cerebellum	R	4.18	4	-82	-22
		Cerebellum	R	4.16	32	-78	-24
		Cerebellum	L	4.06	-18	-76	-18
		Temp occ fusif cor	R	4.06	44	-44	-24
		Cerebellum	L	4.02	-4	-70	-14
		Cerebellum	L	3.89	-8	-64	-14
		Cerebellum	L	3.85	-18	-84	-18
		Cerebellum	L	3.36	-22	-68	-22
		Cerebellum	L	3.32	-26	-66	-24
		Cerebellum	L	3.32	-28	-62	-24
	541	Pari operc cor	R	5.18	60	-22	16
		Cent operc cor	R	4.78	54	-10	10
		Precent gyr	R	4.49	62	-2	16
		Insula	R	4.17	42	-2	6
	326	Cent operc cor	R	4.78	56	6	0
		Planum polare	R	4.72	52	6	-6
		Inf fro gyr p ope	R	4.52	62	20	16
		Inf fro gyr p tri	R	4.43	54	26	20
		Inf fro gyr p ope	R	3.80	58	18	-4
		Mid fro gyr	R	3.73	54	28	28
		Fro orbital cor	R	3.58	52	24	-8

Note: Ext = Extent, L/R = left or right side of the brain, coordinates are given in MNI space. cent = central, cor = cortex, fro = frontal, fusif = fusiform, gyr = gyrus, inf = inferior, lat = lateral, mid = middle, mot = motor, p ope = pars opercularis, occ = occipital, operc = operculum, p tri = pars triangularis, parahipp = parahippocampal, pari = parietal, pos = posterior, sup = superior, supp = supplementary, supram = supramarginal, temp = temporal
